# Antioxidant and Aromatic Properties of Aqueous Extracts of *Pleurotus nebrodensis* as Potential Food Ingredients

**DOI:** 10.3390/foods15020296

**Published:** 2026-01-14

**Authors:** Fortunato Cirlincione, Francesca Vurro, Alexandra-Mihaela Ailoaiei, Saba Shahrivari-Baviloliaei, Graziana Difonzo, Agnieszka Viapiana, Alina Plenis, Antonella Pasqualone, Maria Letizia Gargano

**Affiliations:** 1 Department of Soil, Plant and Foods Science, University of Bari, Via Amendola 165 A, 70126 Bari, Italy; fortunato.cirlincione@uniba.it (F.C.); francesca.vurro97@gmail.com (F.V.); graziana.difonzo@uniba.it (G.D.); antonella.pasqualone@uniba.it (A.P.); marialetizia.gargano@uniba.it (M.L.G.); 2 Department of Analytical Chemistry, Faculty of Pharmacy, Medical University of Gdansk, 80-210 Gdansk, Polandalina.plenis@gumed.edu.pl (A.P.)

**Keywords:** mushroom, chemical composition, antioxidant activity, phenolic compounds, volatile compounds, nutraceutical extract

## Abstract

*Pleurotus nebrodensis* has raised the interest of the food and nutraceutical industry due to its valuable organoleptic characteristics coupled with antibacterial and antitumor properties. Given this interest, this study aimed to identify effective, cheap, and eco-friendly technologies to prepare extracts able to convey the bioactive compounds while retaining the typical mushroom aroma. Two aqueous extracts were prepared based on a freeze–thaw (FT) and ultrasound-assisted (UA) method. The extracts, both in liquid and lyophilized form, were analyzed by HPLC to determine the phenolic compounds. Moreover, the volatile organic compounds, total phenolics, total flavonoids, total phenolic acids, procyanidins, and ascorbic acid were determined, while the antioxidant activity was assessed by DPPH (2,2-diphenyl-1-picrylhydrazyl) and ABTS radical scavenging activity, ferric-reducing/antioxidant power (FRAP), and cupric-reducing antioxidant capacity (CUPRAC) assays. The UA extraction showed significantly higher (*p* < 0.05) phenolics (5.05 vs. 4.02 µg/g DW) and flavonoids (0.74 vs. 0.23 µg/g DW) but lower procyanidins (12.33 vs. 15.93 µg/g DW) and ascorbic acid (6.23 vs. 7.02 µg/g DW) than the FT extracts, resulting in lower antioxidant activity. Among the phenolic constituents, gallic acid was found to be the most abundant in all *P. nebrodensis* extracts. Regarding aroma, FT more effectively preserved volatile alcohols and aldehydes—particularly 1-octen-3-ol and hexanal—while UA led to greater volatile losses. These results highlight that the extraction method significantly affects both antioxidant composition and volatile integrity, with implications for designing *P. nebrodensis*-based food ingredients.

## 1. Introduction

Mushrooms are a widely used and popular food ingredient. They can be used fresh, dried, frozen, or pre-cooked in a variety of traditional and innovative recipes. In addition, mushrooms can be used for preparing alcoholic or aqueous extracts, marketed either fresh or dried, which show promise in the pharmaceutical and nutraceutical fields.

*Pleurotus nebrodensis* (Inzenga) Quèl. [Pleurotaceae, Basidiomycetes] is an edible mushroom native to the Mediterranean environment, growing in association with *Prangos ferulacea* (L.) Lindl [1]. It has been found in some regions of Greece and in the Madonie Mountains in Sicily (Italy), where it is the only fungal species currently reported as endemic [2]. Over the years, the taxonomy of the genus *Pleurotus* has been controversial. This taxon, originally described as *Agaricus nebrodensis* Inzenga, has undergone several changes in synonymy and is recognized as a variety or subspecies within *Pleurotus eryngii*. Molecular analyses conducted on the *P. eryngii* species complex have clarified the taxonomy of these species, which are clearly distinct from other Mediterranean taxa of the genus *Pleurotus* [3]. Furthermore, commercial strains currently available on the international market and erroneously labeled as “*P. nebrodensis*”, especially in China, have been identified as *P. eryngii* subsp. *Tuoliensis* [4].

The good nutritional content, excellent organoleptic characteristics [1], and high consumer appreciation of *P. nebrodensis* have translated into high commercial value, with a consequent risk of overharvesting [4]. This, together with limited distribution and climate change, has led to a decline in wild populations, resulting in its inclusion among the few Italian fungal species currently included on the IUCN (International Union for Conservation of Nature’s) Red List as an endangered species, and in the TOP 50 Mediterranean Island Plants [5]. To conserve the wild population through ex situ conservation [4] and promote and enhance the use of *P. nebrodensis* as a food and medicinal resource, the project “PLEURÒN—Project for the cultivation of *Pleurotus nebrodensis* in a protected environment for food, medicinal and phytogenic purposes”, funded by the Sicilian Regional Administration (Italy), has made it possible to obtain certified mushrooms for use in targeted research on the various activities of the extracts.

In addition to the valuable organoleptic and nutritional characteristics of *P. nebrodensis*, previous studies have demonstrated interesting biological activities, such as antibacterial [6], antitumor, and, recently, antibiofilm [7] properties, allowing it to be defined as a “medicinal mushroom”. Given the interest in *P. nebrodensis* by both the food and nutraceutical industries, it is needed to identify effective, cheap, and eco-friendly technologies to prepare extracts able to convey the bioactive compounds while retaining the typical mushroom aroma. These two elements, crucial for the commercial success of mushroom-based products, can be ensured by using water, a cheap and aroma-free solvent. Other authors have proposed the use of hot water (100 °C) for extracting water-soluble polysaccharides and antioxidants from *P. nebrodensis* [8,9]. However, hot water extraction resulted in lower antioxidant activity compared to non-food solvents, namely acetone and methanol [9]. Furthermore, boiling has been reported to drastically reduce the total concentration of volatile compounds in mushrooms [10]. Therefore, the preparation of aqueous extracts of *P. nebrodensis* at lower temperatures should be investigated by evaluating their antioxidant and aromatic properties, with the aim of assessing their suitability as potential food ingredients.

This study aimed to evaluate the antioxidant and aromatic properties of aqueous extracts of *P. nebrodensis*, both in liquid and lyophilized form, produced using two different eco-friendly protocols based on freeze–thaw and on ultrasound application at 40 °C. Specifically, total phenols, flavonoids, phenolic acids, procyanidins, and ascorbic acid contents were evaluated, as well as the individual phenolic compounds, the antioxidant activities, and the volatile organic compounds.

## 2. Materials and Methods

### 2.1. Mushrooms Material

*P. nebrodensis* basidiomes were produced as part of the ex situ conservation method included in the activities of the “PLEURÒN—Project for the cultivation of *Pleurotus nebrodensis* in a protected environment for food, medicinal, and phytogenic purposes” [4]. Cultivation was carried out using cultivation bags, supplied by Italmiko (Senise, Potenza, Italy), containing a substrate composed of wheat straw and sugar beet residues, and inoculated with a pure culture of *P. nebrodensis* selected strain.

The production process followed standard commercial conditions. It was conducted at the Bergi farmhouse located in Castelbuono (Madonie Mts, northern Sicily) at an altitude of ca. 500 m, by using a tunnel-shaped greenhouse equipped with artificial light, fans, a water mist generator, and an air conditioning unit to set different climatic parameters. During the growth period, the temperature was maintained between 12 and 15 °C, and the relative humidity was close to 98%. Production trials have shown encouraging results in terms of both quality and quantity, as the mushrooms obtained have nutritional and organoleptic characteristics comparable to those of wild mushrooms. Successful cultivation can contribute to the preservation of wild mushrooms in their natural environment by reducing overpicking.

After growing, the basidiomes were collected, finely sliced, and air-dried at room temperature (30 °C) by using stainless steel patented mushroom-drying equipment (Valla, Borgotaro, Parma, Italy). The dried mushrooms were stored at 4 °C in vacuum-sealed bags and, prior to proceeding with the preparation of the extracts, were finely milled with a Vorwerk Bimby^®^ TM7 blender (Vorwerk & Co. KG, Wuppertal, Germany).

### 2.2. Preparation of Aqueous Extracts

Two eco-friendly protocols were applied to prepare the aqueous extracts: (i) the freeze–thaw (FT) protocol of Gargano et al. [11] and (ii) the ultrasound-assisted extraction (UA) protocol of Gogoi et al. [12]. The FT protocol involved the suspension of 3 g of powdered air-dried mushroom (see Section 2.1) in 200 mL of distilled water and then freezing it at −20 °C for 72 h. For the UA protocol, the same amount of powdered air-dried mushroom was suspended in 60 mL of distilled water before being immersed for 14 min in a 200 W ultrasonic bath (Ceia, CP104, Viciomaggio, AR, Italy) with a fixed frequency of 40 kHz, at 40 °C. The two suspensions (FT and UA) were then centrifuged (SL 16R Centrifuge, Thermo Fisher Scientific, Osterode am Harz, Germany) at 4480× *g* for 10 min, and the supernatant was filtered with Whatman 40 paper, obtaining the FT and UA liquid extracts.

Then, an aliquot of both FT and UA liquid extracts was stored at −20 °C until analysis, while another aliquot was lyophilized (Lyovapor^TM^ L-200 Lyophilizer, Flawil, Switzerland) to obtain the FT and UA lyophilized extracts.

The yields of FT and UA protocols were 45.3 and 40.3% (*w*/*w*), respectively.

### 2.3. Chemicals and Instruments

4-Chloro-7-nitrobenzofurazan (NBD-Cl), 2,2-diphenyl-1-picrylhydrazyl (DPPH reagent), 2,2-azinobis (3-ethylbenzothiazoline-6-sulfonic acid) diammonium salt (ABTS reagent), ammonium acetate, neocuproine, and phenolic standards (≥98% purity) were purchased from Sigma-Aldrich (St. Louis, MO, USA). Aluminum chloride was obtained from Across Organics (Morris Plains, NJ, USA) and HPLC-grade acetonitrile (ACN) from J.T. Baker (Center Valley, PA, USA). Other reagents were obtained from POCh (Gliwice, Poland). The redistilled water was prepared by triple distillation of water in a Destmat^®^ Bi-18 system (Heraeus Quarzglas, Hanau, Germany).

The separation and quantification of single phenolic compounds were performed using an HPLC Merck-Hitachi LaChrome device (Darmstadt, Germany). The system consisted of an L-7420 UV-Vis detector, L-7200 autosampler, and L-7360 thermostat. Chromatographic data were collected using a D-7000 HPLC System Manager, ver. 3.1 (Merck-Hitachi, Darmstadt, Germany). The method was validated by linear range, limit of detection (LOD), limit of quantitation (LOQ), precision, and accuracy according to the procedure described previously [13].

The volatile compounds (VOCs) were extracted by headspace solid-phase microextraction (HS-SPME) (Supelco, Bellefonte, PA, USA) using a 75 μm Carboxen/polydimethylsiloxane (CAR/PDMS) SPME fiber (Supelco, Bellefonte, PA, USA) and were analyzed by gas chromatography coupled with mass spectrometry (GC-MS). To ensure the robustness and validation of the results, injections were replicated on two independent GC-MS systems under identical analytical conditions. The first GC-MS system comprised a TRACE 1300 gas chromatograph (Thermo Fisher Scientific, Waltham, MA, USA) and an ISQ Series 3.2 SP1 mass spectrometer (Thermo Fisher Scientific, Waltham, MA, USA), equipped with a VF-WAX MS (Agilent Technologies, Santa Clara, CA, USA) capillary column (60 m length × 0.25 mm internal diameter × 0.25 μm film thickness) and a TriPlus RSH autosampler (Thermo Fisher Scientific, Waltham, MA, USA). The second GC-MS system comprised a gas chromatograph (6850, Agilent Technologies, Santa Clara, CA, USA) coupled with a mass spectrometer (5975, Agilent Technologies, Santa Clara, CA, USA), equipped with a HP-Innowax (Agilent Technologies, Santa Clara, CA, USA) polar capillary column (60 m length × 0.25 mm internal diameter × 0.25 μm film thickness). The identification of compounds was performed with the Xcalibur 2.0 software (Thermo Fisher Scientific), using the NIST (National Institute of Standards and Technology) and Wiley libraries. Only those with a match level above 80% were considered.

### 2.4. Determination of Total Phenolic Compounds (TPC), Total Flavonoid Compounds (TFC), Total Phenolic Acids (TPAC), Total Procyanidins (TAN), and Ascorbic Acid (AA) Content

The TPC was estimated by means of the Folin–Ciocalteu reagent [14]. A measure of 0.2 mL of the Folin–Ciocalteu reagent was added to 0.2 mL of *P. nebrodensis* extract. After 2 min, 2 mL of 7% Na_2_CO_3_ (*w/v*) solution was added. The mixture was agitated and kept at rest for 1 h at room temperature. The absorbance of *P. nebrodensis* extract was measured at 760 nm. Gallic acid solutions were used to obtain a standard curve (linearity range: 0.02–0.25 mg/mL, r = 0.987). The TPC was reported as milligrams of gallic acid equivalents per mL (mg GAE/mL) for liquid extracts and per gram of dry weight (mg GAE/g DW) for lyophilized extracts.

The TFC was estimated according to the European Pharmacopoeia [15]. Briefly, 1 mL of *P. nebrodensis* extract was mixed with 0.1 mL of 5% AlCl_3_ (*w/v*) solution and 1.4 mL of acetic acid and methanol (1:19) mixture. The absorbance was measured after 30 min, at 425 nm. A standard curve (linearity range: 6.5–19.6 µg/mL, r = 0.992) was generated with the use of quercetin solutions. The TFC was reported as milligrams of quercetin equivalents per milliliter (mg QE/mL) for liquid extracts and per gram of dry weight (µg QE/g DW) for lyophilized extracts.

The TPAC was evaluated using Arnov’s reagent [16]. A measure of 1.4 mL of *P*. *nebrodensis* extract was mixed with 0.2 mL of hydrochloric acid (0.5 M), 0.2 mL of Arnov’s reagent, and 0.2 mL of sodium hydroxide solution (1 M). The measurement of absorbance was taken at 490 nm. Caffeic acid solutions were used to obtain a standard curve (linearity range: 4.4–40.3 µg/mL, r = 0.989). The TPAC was expressed as milligrams of caffeic acid equivalents per milliliter (mg CAE/mL) for liquid extracts and per gram of dry weight (mg CAE/g DW) for lyophilized extracts.

The TAN content was estimated using the method developed by Sun et al. [17]. Briefly, 0.5 mL of *P. nebrodensis* extract was mixed with 2.5 mL of vanillin solution (4%, *v*/*v*) and 2.5 mL of hydrochloric acid. After 15 min of incubation, the mixture’s absorbance at 500 nm was measured. Catechin solutions were used to generate a standard curve (linearity range: 0.31–0.85 mg/mL, r = 0.994). The TAN was reported as milligrams of catechin per milliliter (mg GAE/mL) for liquid extracts and per gram of dry weight (mg CE/g DW) for lyophilized extracts.

A slightly modified Abdelmageed method [18] was used for ascorbic acid (AA) determination. Briefly, 0.2 mL of *P. nebrodensis* extract was mixed with 0.2 mL of NaOH (1 M), 0.2 mL of 0.1% (*v*/*v*) 4-chloro-7-nitrobenzofurazane (NBD-Cl) acetone solution, and 1.4 mL of 50% (*v*/*v*) aqueous acetone solution. After 30 min of incubation, an analysis of the mixture’s absorbance was carried out at 582 nm. Ascorbic acid solutions were used to generate a standard curve (linearity range: 0.075–0.726 mg/mL, r = 0.987). The AA content was given as milligrams of AA per milliliter (mg GAE/mL) for liquid extracts and per gram of dry weight (mg AA/g DW) for lyophilized extracts.

### 2.5. HPLC Analysis of Single Phenolic Compounds

The separation of phenolic compounds (phenolic acids—gallic, ferulic, caffeic, *p*-coumaric, vanillic, syringic, and flavonoids—apigenin, kaempferol, rutin, and quercetin) was achieved using a Hypersil Gold C18 column (250 × 4.6 mm, 5 µm, Thermo Scientific, Runcorn, UK) at 30 °C. The mobile phase comprised a mixture of acetonitrile–0.5% acetic acid solution (solvent A) and water–0.5% acetic acid solution (solvent B). A gradient program was as follows: from 0 to 10 min, linear gradient from 5% to 30% solvent A; from 10 to 20 min, linear gradient from 30% to 40% solvent A; from 20 to 30 min, isocratic at 40% solvent A; from 30 to 40 min, linear gradient from 40% to 63% solvent A; and from 40 to 50 min, linear gradient from 63% to 5% solvent A. The injection volume was 20 µL, and the flow rate was 0.8 mL/min. The detection was performed using 280, 320, and 370 nm as preferred wavelengths. The identification of phenolic compounds was based on comparing the retention time and UV-Vis spectrum of their standards. Moreover, a selected sample was spiked with the standard compounds and analyzed again.

The validation parameters for the HPLC procedure are listed in Table 1. The coefficient of variation (CV) values were in the range from 0.35 to 4.21% for intra-day, and from 0.51 to 9.16% for inter-day variations. The data indicate that the precision of the HPLC procedure was acceptable. The stability test showed that the retention CV was lower than 1.8% for the peak area and 0.5% for the retention time. In addition, for the phenolic compounds, the retention times and peak areas were sufficiently stable over 48 h.

### 2.6. Determination of Antioxidant Activity

The DPPH assay was performed using a DPPH radical and compared with Trolox activity [19]. Briefly, 0.1 mL of *P. nebrodensis* extract was added to 2.9 mL of methanolic DPPH solution (100 µmol/L). After 30 min, the absorbance was measured at 517 nm. A standard curve (linearity range: 0.032–0.287 mg/mL, r = 0.986) was obtained using Trolox solutions. The results were given as micromoles of Trolox equivalents per milliliter (µmol TE/mL) for liquid extracts and per gram of dry weight (µmol TE/g DW) for lyophilized extracts.

The ABTS assay was analyzed according to the method of Arnao et al. [20] with some modifications. A measure of 0.1 mL of *P. nebrodensis* extract was added to 2 mL of ABTS solution. The mixture’s absorbance was measured after 6 min at 734 nm. Trolox solutions were used to obtain the standard curve (linearity range: 0.020–0.203 mg/mL, r = 0.983). The results were reported as milligrams of Trolox equivalents per milliliter (mg TE/mL) for liquid extracts and per gram of dry weight (mg TE/g DW) for lyophilized extracts.

The CUPRAC assay was performed according to the method of Apak et al. [21] with some modifications. Briefly, 0.1 mL of *P. nebrodensis* extract was added to 1 mL of neocuprine solution, 1 mL of copper chloride solution, and 1 mL of ammonium acetate buffer solution (pH = 7.00). After 30 min, the mixture’s absorbance was measured at 450 nm. A standard curve (linearity range: 0.024–0.224 mg/mL, r = 0.975) was generated with the use of ascorbic acid solutions. The results were given as milligrams of ascorbic acid equivalents per milliliter (mg AA/mL) for liquid extracts and per gram of dry weight (mg AA/g DW) for lyophilized extracts.

Ferric reducing/antioxidant power (FRAP) assay was determined using the method of Benzie and Strain [22]. Briefly, 0.3 mL of *P. nebrodensis* extract was mixed with 2.3 mL of FRAP. The measurement of absorbance was taken after 30 min at 593 nm. Ferrous sulphate solutions were used to generate a standard curve (linearity range: 0.083–0.436 mg/mL, r = 0.993). The results were reported as millimoles of ferrous sulphate equivalents per milliliter (mmol Fe^2+^/mL) for liquid extracts and per gram of dry weight (mmol Fe^2+^/g DW) for lyophilized extracts.

All antioxidant activity assays were calculated as follows: (a) for liquid extracts A = C × V, where A is the antioxidant activity assay value, C is the concentration of the appropriate standard from the calibration curve in µg/mL, V is the final volume of the extract solution in mL; (b) for lyophilized extracts A = (C × V)/m, where A is antioxidant activity assay value, C is the concentration of the appropriate standard from the calibration curve in µg/mL, V is the final volume of the extract solution in mL and m is the weight of the extract in g.

### 2.7. Determination of Volatile Compounds

The analysis of VOCs of *P. nebrodensis* fruiting bodies, its liquid extracts, and lyophilized forms was carried out by HS-SPME coupled with GC–MS analysis, following the protocol described in Vurro et al. [23].

### 2.8. Statistical Analysis

The results were expressed as the mean ± standard deviation (SD). All analyses were performed in triplicate. The liquid and lyophilized extracts were compared separately, by a 2-sample *t*-test (*p* < 0.05), using Minitab 21 Statistical Software (Minitab Inc., State College, PA, USA).

## 3. Results and Discussion

### 3.1. Bioactive Compounds

When comparing the two extraction protocols, the UA extracts exhibited higher levels of TPC, TFC, and TPAC than the FT extracts, whereas the FT procedure resulted in greater contents of TAN and AA (Table 2). These differences indicate that ultrasound application enhances the release of several phenolic constituents, while the freeze–thawing process better preserves more labile compounds such as TAN and AA (vitamin C). Some studies have shown that US treatment can have both positive and negative effects on the extraction of phenolic compounds. The cavitation effect generated by ultrasonic waves, releasing phenolic compounds and deactivating enzymes related to the degradation of phenolic substances, is related to positive effects [24]. On the other hand, stronger US treatment times and intensities can have adverse effects on phenolic compounds [25]. In this study, the decrease in TAN and AA caused by pretreatments may be related to various factors, such as light, heat, and other factors that, during UA, can cause a decrease in their content [26,27].

As is already known, this is the first report regarding the assessment of TFC, TPAC, TAN, and AA of *P. nebrodensis* extracts. In contrast, various studies have determined the TPC in *P. nebrodensis* and other *Pleurotus* species. Alam et al. [9], who studied the methanolic, acetone, and hot water extracts of Korean *P. nebrodensis* fruiting bodies, recorded a TPC content higher (298 µg/g) than in this study. Rusu et al. [28] reported markedly higher amounts of TPC (from 24.55 to 42.32 mg GAE/g DW) and TFC (from 10.48 to 25.54 mg RE/g DW) in three *Pleurotus* species: *P. eryngii*, *P. ostreatus*, and *P. columbinus* from Romania. The study by Elhusseiny et al. [29] also reported that the amount of TPC in extracts prepared from Egyptian *P. columbinus* was higher (22.50 mg GAE/g DW). Similarly, Rezaeian et al. [30] found higher TPC values in Iranian *P. ostreatus* and *P. eryngii* (0.37 and 2.5 mg GAE/g DW, respectively). These discrepancies highlight how species, substrate, developmental stage, and extraction method strongly influence the phenolic content of mushroom extracts.

### 3.2. Single Phenolic Compounds

**Figure 1:**
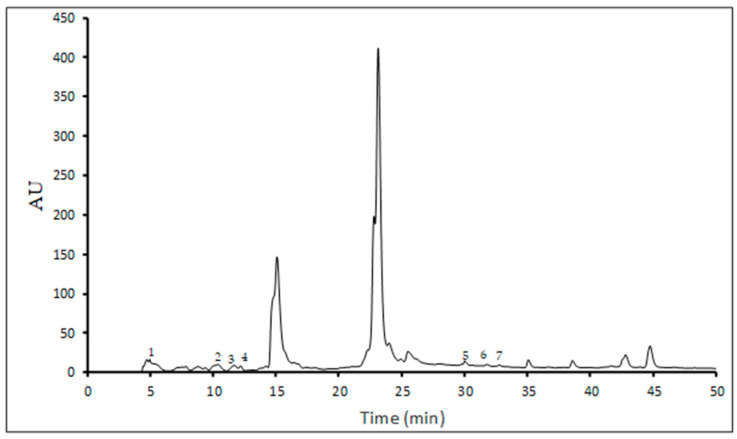
HPLC phenolic profile of lyophilized UA extract (peak 1-gallic acid, peak 2-caffeic acid, peak 3-*p*-coumaric acid, peak 4-ferulic acid, peak 5-rutin, peak 6-quercetin, peak 7-kaempferol).

In this study, ten single phenolic compounds were identified and quantified, including six phenolic acids (gallic, ferulic, caffeic, *p*-coumaric, vanillic, and syringic acids) and four flavonoids (apigenin, kaempferol, rutin, and quercetin) (Table 3), while Figure 1 shows a representative chromatogram of the lyophilized UA extract.

Generally, the UA extraction showed a higher content of phenolic compounds than the FT protocol. The selection of ultrasonic pretreatment parameters and drying conditions strongly influences the content of phenolics of edible mushrooms [31]. However, there was no significant difference in the analyzed constituents between extracts obtained by the FT and UA methods, except for gallic acid, *p*-coumaric acid, and quercetin in the liquid extract, and gallic acid and *p*-coumaric acid in lyophilized extracts. In addition, gallic acid was the main phenolic compound identified in *P. nebrodensis* extracts, while vanillic and syringic acids and apigenin were not found in the analyzed samples. Interestingly, kaempferol was found exclusively in the UA-derived extracts, indicating that ultrasound treatment may enhance the release or detection of specific flavonoids.

The variability in phenolic composition reported in the literature for *Pleurotus* spp. reflects the influence of genetic, environmental, and methodological factors. Within this context, the present work provides the first detailed characterization of the individual phenolic constituents of *P. nebrodensis* aqueous extracts. Gallic acid was the most abundant phenolic compound in all the extracts. In the literature data regarding *Pleurotus* species, Palacios et al. [32] and Jabłonska-Rys et al. [33] reported a higher amount of gallic acid in *P. ostreatus* (290.34 and 333.37 µg/g DW). Radzki et al. [34] found a caffeic acid content of 0.12 µg/g DW for *P. ostreatus*, and Matkovits et al. [35] detected the presence of caffeic acid in the range between 0.01 and 1.54 µg/g DW in *P. ostreatus*. Gasecka et al. [36] found 7.17 and 9.12 µg/g DW of *p*-coumaric acid in *P. ostreatus*, and 6.49 and 8.00 µg/g DW in *P. eryngii*, while Reis et al. [37] reported lower amounts of this phenolic acid, with values 0.81 µg/g DW for *P. ostreatus* and 1.04 µg/g DW for *P. eryngii*. In addition, Radzki et al. [34] determined 0.09 and 0.10 µg/g DW of syringic acid in *P. eryngii* and *P. ostreatus*, respectively, while Matkovits et al. [35] found a lower amount (<0.05 µg/g DW) of syringic acid in *P. ostreatus*. In the case of rutin, Vamanu [38] did not find this compound in *P. ostreatus*, although Jayakumar et al. [39] recorded a content of 31.20 mg rutin/100 g. For *P. ostreatus* and *P. eryngii*, Akyüz et al. [40] reported a quercetin content of 0.25 µg/g DW.

An overall comparison of *P. nebrodensis* with other *Pleurotus* species shows that it is poorer in gallic acid, *p*-coumaric acid, and rutin, while the amount of caffeic acid and quercetin is on the same level.

### 3.3. Antioxidant Activity

In this study, the antioxidant activity was measured with the use of four tests: DPPH, ABTS, FRAP, and CUPRAC. The selection of different methods allows a better understanding of the wide variety and range of actions of antioxidant compounds present in *P. nebrodensis*. According to the results (Table 4), in general, the FT extracts showed higher antioxidant activity than UA extracts, except for the values determined by the CUPRAC assay. The freezing–thawing process seems to have a beneficial effect on antioxidant potential. The efficacy of antioxidant compounds depends on several factors, such as chemical structure, temperature, concentration, and the presence of synergistic effects. Since each method is based on a different chemical system and/or reaction, different results of *P. nebrodensis* could be expected depending on the method used. The observed differences in this study were significant (*p* < 0.05), except for FRAP values found in lyophilized extracts. Higher antioxidant activity of extracts prepared by FT may be the result of the higher presence of AA and TAN compared to the UA extracts.

To the best of our knowledge, this is the first report of the comparison of the antioxidant activity of *P. nebrodensis* extracts determined with four different assays, including the ABTS and CUPRAC assays.

The literature on the antioxidant activity of *P. nebrodensis* is very scarce, and only DPPH and FRAP assays have been determined so far [9]. Although we also included these tests in our study, the recorded DPPH and FRAP values cannot be compared with those obtained from literature screening due to the differences in the units. For example, Alam et al. [9] analyzed hot water extracts of *P. nebrodensis* fruiting bodies and, at the sample concentration of 8 mg/mL, found DPPH values of 1.817 mg/mL (IC50), while the reducing power was 1.817 mg/mL (IC50).

In *P. ostreatus*, the IC50 index values were 8.88 mg/mL [41], while Elhusseiny et al. [29] analyzed *P. columbinus* and found an IC50 value of 35.13 mg/mL. In contrast, Angelini et al. [42] obtained lower DPPH values, 2.25 and 4.98 mg/mL (EC50), for *P. columbinus* extracts, while Rusu et al. [28] found DPPH at the levels of 9.82, 11.48, and 14.26 mg/mL (IC50) for *P. ostreatus*, *P. eryngii*, and *P. columbinus*, respectively. In addition to the known effect of the species, according to the literature, the different conditions, such as temperature and time of extraction, could influence the release efficiency of specific antioxidant compounds, such as phenolic compounds, and regulate the antioxidant capacity of the final extract.

### 3.4. Volatile Compounds

**Figure 2:**
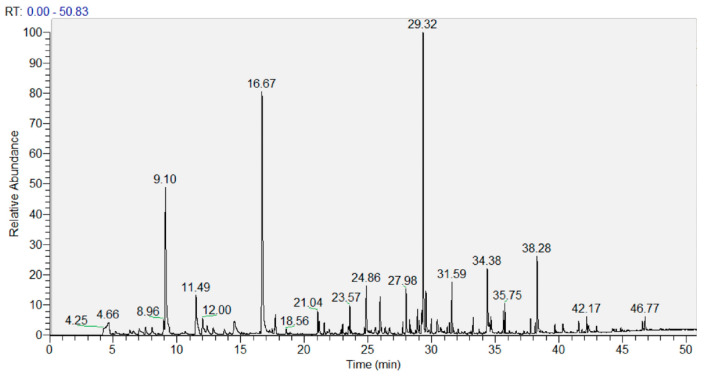
Representative GC-MS chromatogram of the lyophilized ultrasound-assisted (UA) extract.

The analysis of volatile compounds (VOCs) was performed using GC-MS. As a representative chromatogram, Figure 2 shows that of the lyophilized UA extract. The resolution was good, and the peaks were very neat. Chromatograms of a similar quality were obtained for all samples analyzed.

Both the starting *P. nebrodensis* fruiting bodies and their extracts revealed a complex profile, with several key chemical classes, predominantly alcohols and aldehydes, but also ketones, carboxylic acids, alkanes, furans, pyrazines, aromatic compounds, and sulfur compounds (Table 5). The volatile profile of edible mushroom species, in fact, can be extremely rich, with over 100 compounds identified [43,44].

Several compounds are particularly associated with the distinctive mushroom-like and earthy odors of *Pleurotus* spp., including 1-octen-3-ol, 1-octanol, 3-octanone, 1-octen-3-one, octanol, and 1-pentanol [47,48]. 1-Octen-3-ol and 3-octanone are typically found in high concentrations in edible fungi and are the principal components of the volatile profile [47]. Zhang et al. [49] have demonstrated a positive correlation between these two compounds and the antioxidant activity in *Volvariella volvacea* and *P. ostreatus* mushrooms during maturity. Yin et al. [50], focusing on the comparison of six *Pleurotus* species, identified 1-octen-3-one and 1-octen-3-ol as key odor compounds in *P. citrinopileatus*, *P. djamor*, *P. ostreatus*, *P. floridanus*, and *P. sapidus*, whereas 1-octen-3-one, 1-octen-3-ol, and 2-octenal were observed in *P. cornucopiae*. 1-Octen-3-ol was found to be the most represented volatile compound in different strains of *P. eryngii*, followed by hexanal, 3-octanone, and 2-octenal [51].

In the present study, 1-octen-3-ol in particular, but also 1-octanol and 1-pentanol, were consistently detected in both the mushroom fruit bodies and all the extracts, confirming their key role in shaping the sensory identity of *Pleurotus* mushrooms [47,48]. The C8 aliphatic compounds originate from the enzymatic degradation of linoleic acid and other unsaturated fatty acids and are central to fungal aroma biosynthesis [52]. In fact, although mushrooms are primarily composed of water and contain relatively low amounts of total lipids (1–8%), linoleic acid is the most abundant and predominant fatty acid in many fungal species, including those of the *Pleurotus* genus [53,54,55].

Aldehydes were the most numerous among the various classes of compounds detected, second only to alcohols in abundance. Among them, benzaldehyde, hexanal, and 3-methylbutanal were particularly concentrated in the mushroom fruit bodies, followed by benzenacetaldehyde, nonanal, and octanal. Benzaldehyde is produced from phenylalanine catabolism and the degradation of benzoic acid [56], while hexanal, octanal, and nonanal are products of the oxidative degradation of polyunsaturated and monounsaturated fatty acids, probably due to the prolonged air-drying at room temperature carried out at the producing company that provided the mushrooms. 3-Methylbutanal, instead, considering that edible mushrooms are also a rich source of enzymes [57], could be formed from leucine through a series of enzymatic processes catalyzed by transaminase and dehydrogenase [58,59]. These aldehydes are widely recognized as significant contributors to the characteristic aroma of mushrooms [44]. Ketones, such as 2-octanone and 2-methyl-3-octanone, were also identified, contributing characteristic mushroom and cheese-like notes. Together with alcohols, these ketones are known to interact synergistically, enhancing flavor intensity [60].

The extraction procedure can influence the volatile profile, leading to chemical and physical changes responsible for the degradation and loss of some compounds or the production of new ones, either volatiles or non-volatiles. In fact, several compounds with distinct aromatic notes, such as hexanoic acid (fatty, cheesy), phenylethyl alcohol (floral), and 5-hepten-2-one, 6-methyl (fruity), were present in the starting mushrooms but were absent in the aqueous extracts and lyophilized powders. This suggested that these compounds were either highly volatile or not sufficiently water-soluble to be effectively recovered through aqueous extraction.

To better contextualize the results, it should be noted that the volatile compounds from *Pleurotus* species have been studied mainly focusing on organic extracts (e.g., prepared with methanol, ethanol, hexane), rather than aqueous extracts. In fact, while aqueous extracts have been extensively studied for their health-promoting components (antioxidants, antimicrobials, prebiotics) [9,61], their aroma has been little investigated and, in particular, no data are available on *P. nebrodensis* extracts. One study analyzed the characteristic odorants contained in the oil of *P. eryngii* var. *tuoliensis*, which is sometimes mistakenly labeled as *P. nebrodensis*, obtained by hydrodistillation. Methional, 1-octen-3-ol, and nonanal were identified as the main aroma-active components of this oil [62]. In contrast, while 1-octen-3-ol was detected at very high levels both in the starting *P. nebrodensis* fruiting bodies and their FT and UA extracts, nonanal was not one of the main compounds detected, and methional was absent.

The two different extraction protocols used to prepare aqueous extracts had an impact on the composition of VOCs, with the FT protocol generally showing a higher ability to retain volatiles compared to the UA extraction, despite the latter using a higher sample to water ratio (3:60 instead of 3:200). In fact, a noticeable difference in favor of the FT method can be observed by comparing the sums of the single classes of chemical compounds, especially alcohols and aldehydes, as well as the most abundant individual compounds, particularly 1-octen-3-ol and hexanal. The higher presence of the latter two compounds, known to be products of both enzymatic and non-enzymatic lipid oxidation, was consistent with the lower amounts of specific antioxidants such as TPC, and, in particular, gallic acid, detected in the FT extract. Interestingly, the FT extract contained a higher amount of ascorbic acid than the UA extract, but the increase in hexanal was not effectively prevented by this antioxidant, as already reported by other authors [63].

In terms of qualitative profile, the extracts generally showed the same compounds detected in the original mushrooms, albeit with variations in concentration, but no newly formed volatile compounds were detected. A marked decrease in benzaldehyde and other related compounds (phenylethyl alcohol, benzeneacetaldehyde, 2-methylbenzeneacetaldehyde) was observed with both the extraction methods, even in the lyophilized forms of the extracts, probably due to the easy oxidation to non-volatile benzoic acid and derivatives.

3-Methylbutanal was the only compound present in significantly higher amounts in the extract obtained with the UA method, compared to the FT protocol. Considering the enzymatic origin of this aldehyde [56,57], the difference in 3-methylbutanal concentration between the two extraction methods can be attributed to the different temperatures applied, as temperature can significantly affect enzymatic activity [64]. In fact, UA extraction, carried out at 40 °C, may have favored enzymatic activity compared to FT extraction, which was conducted at −20 °C.

The choice of the subsequent drying method, needed to obtain a powdery extract, is also critical, as it determines whether volatile constituents are retained or lost. Compared to other drying techniques, lyophilization was chosen because other authors reported that, both in mushrooms and in other vegetable products, it resulted in minimal reductions in volatile concentrations [65,66,67]. In the present study, the total sum of all chemical classes detected in the mushroom fruiting bodies decreased by about 11% in the lyophilized form of the FT extracts, while a decrease of 33% was observed in the lyophilized form of the UA extracts. In contrast, some compounds identified in the starting mushrooms at relatively low amounts and not detected at all in the liquid extracts (such as 1-octen-3-ol, 3,5-dimethoxybenzaldehyde, 2-nonenal, 2,4-nonadienal) were observed in their lyophilized forms, likely because of a concentration effect associated with the removal of water. However, the cost of lyophilization and the need for specific equipment can limit its use, although this technique is known to be effective in preserving nutrients and bioactive compounds [43].

The aroma of mushroom-based products is crucial for their commercial success, the odor notes of mushrooms being either desirable or undesirable, depending on the final application. For products where a strong mushroom aroma is needed, such as flavoring powders, it is essential to use extraction protocols able to preserve compounds like 1-octen-3-ol, which is recognized as responsible for the “earthy” and “mushroom-like” odor [10]. So, the FT protocol, followed by lyophilization, is the most advisable in this case. In contrast, when the mushroom aroma is less important or even undesired, as could be the case with specific nutraceutical supplements, the UA extraction, able to retain a much lower total amount of VOCs, as well as the liquid form of the extract, more diluted, are the most suitable options. This aspect must be carefully considered when developing mushroom extracts to be proposed as potential food ingredients, where sensory characteristics are fundamental for consumer acceptance [68,69].

## 4. Conclusions

The extraction method markedly influenced the chemical composition, antioxidant capacity, and volatile profile of *P. nebrodensis* extracts. UA extraction resulted in higher levels of TPC and TFC, whereas the FT method preserved greater amounts of TAN and AA, which contributed to overall higher antioxidant performance. Gallic acid was consistently identified as the predominant phenolic compound across all extract types.

The FT protocol was more effective in retaining key aroma-active compounds essential for the characteristic mushroom-like odor, whereas the UA method led to greater losses of volatiles. Lyophilization contributed to the partial concentration of specific volatiles but also caused reductions depending on the extraction method used.

*P. nebrodensis* extracts are promising functional or aromatic ingredients, but future research should expand the range of extraction technologies, explore solvent modulation, assess sensory and bioavailability outcomes, and investigate the stability of both antioxidant and volatile fractions in real food matrices.

## Figures and Tables

**Table 1:** Validation parameters of the calibration curves for analytes quantified in this study (*n* = 3).

Analytes	Regression Equation ^a^	Linearity (µg/mL)	r	LOD (µg/mL)	LOQ (µg/mL)
Gallic acid	y = 47,383x − 88,823	10.4–132.5	0.988	1.6	3.9
Ferulic acid	y = 193,773x + 438,086	11.2–126.3	0.983	2.1	9.9
Caffeic acid	y = 68,856x + 453,666	12.0–87.4	0.992	2.8	10.3
*p*-Coumaric acid	y = 189,934x − 146,344	11.5–81.3	0.988	2.9	8.1
Vanillic acid	y = 58,832x + 73,392	25.3–185.7	0.991	8.1	23.8
Syringic acid	y = 51,227x + 68,443	9.6–79.0	0.986	4.2	13.6
Apigenin	y = 24,288x − 24,022	10.2–85.44	0.986	2.1	5.8
Kaempferol	y = 89,959x − 333,649	10.6–104.3	0.982	2.5	7.7
Rutin	y = 25,883x − 34,866	13.8–100.6	0.988	3.5	13.1
Quercetin	y = 38,566x − 232,282	12.8–119.6	0.985	3.1	10.2

^a^ y is the peak area; x refers to the concentration of compounds (µg/mL); LOD—limit of detection; LOQ—limit of quantification.

**Table 2:** Total phenolics (TPC), flavonoids (TFC), phenolic acids (TPAC), procyanidins (TAN), and ascorbic acid (AA) of *P. nebrodensis* extracts. FT = freeze–thaw extraction method; UA = ultrasound-assisted extraction method.

	Liquid Extract (µg/mL)		Lyophilized Extract (µg/g DW)	
	FT	UA	FT	UA
TPC	14.69 ± 0.24 b	17.83 ± 0.08 a	4.02 ± 0.12 b	5.05 ± 0.07 a
TFC	0.82 ± 0.06 b	2.28 ± 0.24 a	0.23 ± 0.07 b	0.74 ± 0.12 a
TPAC	0.96 ± 0.02 b	1.35 ± 0.05 a	0.28 ± 0.01 a	0.34 ± 0.03 a
TAN	40.53 ± 3.59 a	23.16 ± 1.41 b	15.93 ± 2.54 a	12.33 ± 1.10 b
AA	23.37 ± 1.44 a	20.39 ± 0.79 b	7.02 ± 1.29 a	6.23 ± 0.54 a

Different letters in the same row indicate significant differences at *p* < 0.05, considering the comparison between the two liquid extracts and the two lyophilized extracts, separately.

**Table 3:** Content of individual phenolic compounds in *P. nebrodensis* extracts. FT = freeze–thaw extraction method; UA = ultrasound-assisted extraction method.

Compound	Liquid Extract (µg/mL)		Lyophilized Extract (µg/g DW)	
	FT	UA	FT	UA
Gallic acid	268.62 ± 1.38 b	317.04 ± 3.46 a	92.48 ± 2.06 b	103.24 ± 2.97 a
Ferulic acid	3.15 ± 0.69 a	4.08 ± 0.76 a	0.89 ± 0.10 a	1.30 ± 0.53 a
Caffeic acid	3.99 ± 0.54 a	3.75 ± 0.34 a	1.12 ± 0.88 a	1.32 ± 0.45 a
*p*-Coumaric acid	1.62 ± 0.09 a	0.69 ± 0.03 b	0.48 ± 0.5 a	0.24 ± 0.05 b
Vanillic acid	n.d.	n.d.	n.d.	n.d.
Syringic acid	n.d.	n.d.	n.d.	n.d.
Apigenin	n.d.	n.d.	n.d.	n.d.
Kaempferol	n.d.	0.99 ± 0.08	n.d.	0.23 ± 0.11
Rutin	2.04 ± 0.14 a	2.49 ± 0.10 a	0.73 ± 0.15 a	0.81 ± 0.21 a
Quercetin	0.63 ± 0.05 b	1.36 ± 0.11 a	0.25 ± 0.09 a	0.34 ± 0.08 a
Total	280.05	330.42	95.95	107.49

Different letters in the same row indicate significant differences at *p* < 0.05, considering the comparison between the two liquid extracts and the two lyophilized extracts, separately; n.d.—not detected.

**Table 4:** Antioxidant activity of *P. nebrodensis* extracts. FT = freeze–thaw extraction method; UA = ultrasound-assisted extraction method.

	Liquid Extract (Values per mL)		Lyophilized Extract (Values per mg DW)	
	FT	UA	FT	UA
DPPH (µmol TE)	0.41 ± 0.03 a	0.10 ± 0.00 b	0.12 ± 0.01 a	0.01 ± 0.00 b
ABTS (mg TE)	10.56 ± 0.12 a	5.02 ± 0.04 b	3.34 ± 0.20 a	1.02 ± 0.06 b
CUPRAC (mg AA)	6.33 ± 0.15 b	8.31 ± 0.23 a	2.12 ± 0.18 b	3.01 ± 0.27 a
FRAP (mmol Fe^2+^)	12.45 ± 0.63 a	9.36 ± 0.16 b	4.04 ± 0.97 a	3.22 ± 0.14 a

Different letters in the same row indicate significant differences at *p* < 0.05, considering the comparison between the two liquid extracts and the two lyophilized extracts, separately.

**Table 5:** Volatile compounds of *P. nebrodensis* air-dried mushroom and its extracts. RT = retention time; FT = freeze–thaw extraction method; UA = ultrasound-assisted extraction method.

Compound	RT (min)	Air-Dried Mushroom (μg/g DW)	Liquid Extract		Lyophilized Extract		Odor Notes ^(1,2)^
			FT (μg/mL)	UA (μg/mL)	FT (μg/g DW)	UA (μg/g DW)	
Carboxylic acids							
Acetic acid	29.55	53.51 ± 8.37	4.66 ± 1.12 a	2.53 ± 0.45 a	50.05 ± 2.91 a	44.68 ± 4.26 a	Sharp, pungent, sour, vinegar
Hexanoic acid	38.28	88.19 ± 4.99	n.d.	n.d.	n.d.	n.d.	Fatty, cheesy, floral
Alcohols							
Z,Z-2,5-Pentadecadien-1-ol	19.84	0.30 ± 0.03	0.80 ± 0.24 a	0.41 ± 0.06 a	0.76 ± 0.01a	0.56 ± 0.18 a	Not specified
1-Pentanol	23.46	52.21 ± 2.12	1.81 ± 0.11a	1.38 ± 0.24 a	18.20 ± 1.95a	11.15 ± 0.86 b	Sweet
6-Methyl-cyclohex-2-en-1-ol	23.94	0.16 ± 0.28	n.d.	n.d.	4.88 ± 0.46a	1.22 ± 0.17 b	Herbal, green
1-Hexanol	26.7	75.25 ± 2.10	1.08 ± 0.03 a	1.36 ± 0.53 a	7.06 ± 0.33 b	8.96 ± 0.60 a	Pungent, ethereal, fusel oil, fruity and alcoholic, sweet with a green top note
3,5-Octadien-2-ol	28.39	8.75 ± 0.25	n.d.	n.d.	7.20 ± 0.17 a	5.37 ± 0.95 b	Green, fresh
1-Octen-3-ol	29.32	844.00 ± 23.77	426.69 ± 6.24 a	154.94 ± 3.53 b	708.47 ± 14.16 a	296.46 ± 16.21 b	Earthy, metallic, mushroom-like, vegetal, cabbage
1-Hexanol, 2-ethyl	30.44	13.22 ± 0.25	4.38 ± 0.38 a	4.44 ± 0.18 a	13.01 ± 0.86 b	21.00 ± 1.39 a	Citrus, fresh, floral, oily, sweet
1-Octanol	32.08	30.41 ± 3.89	1.36 ± 0.03 a	0.75 ± 0.01 b	4.69 ± 0.12 b	7.24 ± 0.33 a	Waxy, green, orange, floral, fruity
Phenylethyl alcohol	39.93	10.59 ± 0.62	n.d.	n.d.	n.d.	n.d.	Fruity, floral, sweet
Aldehydes							
Butanal, 2-methyl	8.96	11.71 ± 1.63	1.05 ± 0.12 a	0.85 ± 0.01 a	18.01 ± 0.10 a	17.21 ± 1.93 a	Musty, chocolate, nutty, malty, fermented
Butanal, 3-methyl	9.10	101.12 ± 10.2	9.22 ± 0.52 b	17.10 ± 1.55 a	105.08 ± 2.88 b	238.77 ± 5.91 a	Ethereal, aldehydic, chocolate, peach, fatty
3,5-Dimethoxybenzaldehyde	9.67	3.12 ± 0.38	n.d.	n.d.	2.85 ± 0.18 a	2.31 ± 0.05 b	Fruity
Pentanal	11.49	21.54 ± 1.95	8.46 ± 0.14 a	5.27 ± 0.32 b	76.25 ± 3.02 a	81.93 ± 2.65 a	Fermented, fruity, berry nuances
Hexanal	16.67	274.73 ± 0.12	43.18 ± 0.84 a	23.28 ± 0.77 b	547.7 ± 10.24 a	450.64 ± 18.69 b	Green, fatty, leafy, vegetal, fruity, woody nuance
Heptanal	18.56	1.91 ± 0.44	1.61 ± 0.20 a	1.79 ± 0.16 a	20.33 ± 0.73 a	21.72 ± 1.53 a	Fatty, green, citrus
4,4-Dimethylpent-2-enal	23.11	0.84 ± 0.13	n.d.	n.d.	6.68 ± 0.58 a	1.30 ± 0.72 b	Sweet, floral, cocoa
Octanal	24.86	23.93 ± 0.31	14.92 ± 0.19 a	11.37 ± 0.29 b	137.74 ± 2.73 a	57.16 ± 0.00 b	Aldehydic, waxy, citrus, with a green peel nuance
2-Heptenal, (Z)-	25.97	12.39 ± 0.74	2.77 ± 0.09 a	0.72 ± 0.14 b	42.15 ± 2.99 a	9.08 ± 0.00 b	Green, fatty, leafy, vegetal, fruity, woody nuance
Nonanal	27.98	39.92 ± 1.70	7.72 ± 1.20 a	8.60 ± 0.86 a	56.28 ± 3.07 a	54.95 ± 0.27 a	Waxy, aldehydic, citrus, fresh green lemon peel, cucumber
2-Octenal, (E)-	29.01	9.43 ± 0.37	2.26 ± 0.21 a	1.06 ± 0.08 b	70.78 ± 2.36 a	10.48 ± 0.82 b	Fatty, green, herbal
Benzaldehyde	31.59	310.29 ± 34.57	2.59 ± 0.49	2.63 ± 0.04	40.05 ± 0.32 a	35.63 ± 0.44 b	Almond, fruity, nutty
2-Nonenal, (Z)-	31.71	10.73 ± 0.84	n.d.	n.d.	11.98 ± 0.46 a	4.97 ± 0.06 b	Floral, fatty, waxy, cucumber
Benzeneacetaldehyde	34.38	67.03 ± 1.57	1.50 ± 0.04	n.d.	2.58 ± 0.89 a	1.68 ± 0.22 b	Spice
Benzenealdehyde, 2-methyl	34.53	13.72 ± 0.20	0.30 ± 0.09 b	1.58 ± 0.05 a	n.d.	n.d.	Sweet, floral
2,4-Nonadienal, (E,E)-	35.51	10.26 ± 1.23	n.d.	n.d.	4.78 ± 0.48 a	1.55 ± 0.29 b	Fatty, melon, waxy, green, leaf, cucumber, tropical fruit
Benzenaldehyde, 3,5-dimethyl	38.00	n.d.	1.29 ± 0.19 a	1.23 ± 0.06 a	0.91 ± 0.02 a	0.53 ± 0.01 b	Fruity
Alkanes							
Decane	12.35	111.36 ± 0.22	4.53 ± 0.67	n.d.	8.39 ± 6.72 b	36.07 ± 2.26 a	Sweet, grassy, waxy, ethereal, oily, citrus, fresh, fish, musty
Octadecane, 6-methyl	17.16	2.51 ± 0.86	0.93 ± 0.13 a	0.95 ± 0.14 a	4.06 ± 0.08 a	3.98 ± 0.45 a	Not specified
Dodecane	21.58	13.71 ± 0.19	n.d.	n.d.	4.81 ± 0.49 b	20.5 ± 1.69 a	Gasoline-like
Pentadecane	23.01	8.07 ± 0.75	6.75 ± 0.60 a	6.23 ± 0.56 a	11.45 ± 0.57 a	10.39 ± 0.26 a	Waxy
Heptacosane	25.58	n.d.	4.73 ± 0.19 a	2.72 ± 0.11 b	7.69 ± 0.47 b	12.23 ± 0.10 a	Waxy
2-Octen, 2-butyl	34.71	18.51 ± 1.67	n.d.	n.d.	n.d.	n.d.	Green, fruity, metallic, oily, tropical, fatty, sweaty, goat
Ketones							
2-Pentanone	9.52	2.43 ± 0.24	n.d.	n.d.	2.78 ± 0.14	2.25 ± 0.18	Ethereal, sweet banana, fermented, woody
2-Hexanone	12.00	22.07 ± 0.23	0.88 ± 0.10	n.d.	32.63 ± 1.24 a	31.39 ± 1.68 a	Fruity, fungal, buttery
2-Hexanone, 4-methyl	21.04	12.52 ± 1.01	n.d.	0.66 ± 0.06	16.35 ± 0.67 b	21.94 ± 1.47 a	Sweet, fruity, floral, green, herbal, dairy
3-Heptanone, 5-methyl	23.67	9.42 ± 0.20	1.79 ± 0.08 a	0.88 ± 0.06 b	4.50 ± 0.18 a	3.36 ± 0.04 b	Herbal, sweet, oily
3-Octanone, 2-methyl	25.94	28.54 ± 0.52	1.80 ± 0.12 a	0.83 ± 0.09 b	49.98 ± 1.97 a	25.20 ± 1.61 b	Musty, mushroom, moldy, cheesy, fermented, green, vegetative
2-Octanone	24.71	11.08 ± 0.17	1.48 ± 0.03	n.d.	5.54 ± 0.29 b	7.32 ± 0.24 a	Musty, bleu cheese, mature cheese
5-Hepten-2-one, 6-methyl	26.35	5.41 ± 0.06	n.d.	n.d.	n.d.	n.d.	Fruity, apple, creamy, cheese, banana
2-Heptanone, 4,6-dimethyl	26.46	3.61 ± 0.28	n.d.	n.d.	n.d.	n.d.	Fruity, fatty
3-Methyl-3-cyclohexen-1-one	29.22	19.38 ± 1.12	n.d.	n.d.	n.d.	n.d.	Sweet, nutty, walnut, fruity, almond
2-Undecanone	33.10	9.05 ± 0.84	0.49 ± 0.07	n.d.	3.61 ± 0.10 a	1.86 ± 0.19 b	Waxy, fruity, pineapple
5,9-Undecadien-2-one, 6,10-dimethyl-, (E)-	38.55	4.11 ± 0.05	n.d.	n.d.	n.d.	n.d.	Floral
Aromatic compounds							
Benzene, 1,3-bis(1,1-dimethylethyl)-	28.88	22.11 ± 1.28	3.42 ± 0.09 b	5.25 ± 0.03 a	3.00 ± 0.14 b	22.72 ± 0.71 a	Not specified
3,5-Dihydroxytoluene	31.88	4.10 ± 0.15	n.d.	n.d.	n.d.	n.d.	Not specified
Benzeneamine, 3-methyl	37.78	14.63 ± 0.45	n.d.	n.d.	9.31 ± 0.81 a	10.51 ± 0.77 a	Ammoniacal and fish-like
Lactone							
2(3H)-Furanone, dihydro-3-methylene	35.62	34.94 ± 2.62	5.62 ± 0.05	n.d.	43.4 ± 0.87 a	23.95 ± 0.37 b	Green, vegetal
Furan							
Furan, 2-pentyl	22.90	24.96 ± 2.48	n.d.	n.d.	24.35 ± 1.79 a	4.07 ± 0.42 b	Fruity, green, beany, vegetable
Pyrazine							
Pyrazine, 2,6-dimethyl	26.06	3.33 ± 0.28	n.d.	n.d.	15.21 ± 0.39 a	5.17 ± 0.10 b	Cocoa, roasted nuts, roast beef, coffee
Sulfur compounds							
Mercaptoacetic acid, bis(trimethylsilyl)-	27.74	4.91 ± 0.10	7.64 ± 0.35 a	7.86 ± 0.11 a	8.90 ± 0.48 a	6.63 ± 0.25 b	Sulfuric odor
Propanal, 3(methylthio)-	29.81	3.21 ± 0.13	n.d.	0.61 ± 0.07	6.41 ± 0.50 a	4.72 ± 0.37 b	Boiled potato
Dimethyl sulfone	39.77	0.65 ± 0.04	n.d.	n.d.	3.25 ± 0.17 a	2.07 ± 0.23 b	Sulfuric odor, burnt
Sums of chemical classes							
Carboxylic acids		141.7 ± 13.36	4.66 ± 1.12 a	2.53 ± 0.45 a	50.05 ± 2.91 a	44.68 ± 4.26 a	
Alcohols		1034.89 ± 33.11	436.12 ± 7.03 a	163.28 ± 4.55 b	764.27 ± 18.05 a	351.96 ± 20.69 b	
Aldehydes		912.67 ± 56.38	96.87 ± 4.32 a	75.48 ± 4.33 b	1144.15 ± 31.05 a	989.91 ± 33.59 b	
Alkanes		154.16 ± 3.69	16.94 ± 1.59 a	9.9 ± 0.81 b	36.4 ± 8.33 b	83.17 ± 4.76 a	
Aromatic compounds		40.84 ± 1.43	3.42 ± 0.09 b	5.25 ± 0.03 a	12.31 ± 0.95 b	33.23 ± 1.48 a	
Ketones		127.62 ± 4.72	6.44 ± 0.4 a	2.37 ± 0.21 b	115.39 ± 4.59 a	93.32 ± 5.41 b	
Sulfur compounds		8.77 ± 0.27	7.64 ± 0.35 b	8.47 ± 0.18 a	18.56 ± 1.15 a	13.42 ± 0.85 b	
Total sum		2420.65 ± 112.96	572.09 ± 14.90 a	267.28 ± 10.56 b	2141.13 ± 67.03 a	1609.69 ± 71.04 b	

Data are expressed as mean ± SD. Different letters in the same row indicate significant differences at *p* < 0.05, considering the comparison between the two liquid extracts and the two lyophilized extracts, separately. ^1,2^ according to [45,46]. n.d.—not detected.

## Data Availability

The original contributions presented in this study are included in the article. Further inquiries can be directed to the corresponding author.
